# A Reform Needed

**Published:** 1875-04

**Authors:** 


### A REFORM NEEDED.

The sanitary reasons for discarding the old system of scavenging are apparent. The air of densely populated localities is nightly filled with the poisonous and disgusting odors from the contents of sinks in process of removal; and low forms of fevers, diarrhœa, dysenteries, &c., are thereby created or seriously aggravated. During the warm months there has been at times such an increase of these diseases in New York, that the sanitary authorities have entirely prohibited the business until colder weather.

Of the new and improved methods for the removal of night-soil from privies and sinks, of which there are not less than six, only one has yet been practically introduced in this city. That other acceptable methods are not now in use is due to the obstinate adherence to the old system by those engaged in the business of scavenging, and their strenuous opposition to all improvements. The Board of Health welcomes and encourages any device or apparatus by which the necessary work can be accomplished without offence, and to all such afford equal facilities and privileges. The new and improved process above referred to consists of a powerful pump with a four-inch hose, by which the night-soil is conveyed from the privy to an air-tight tank. The only alleged objections to the introduction of new and improved methods which seem to demand an answer, are, that the laboring men employed under the old system will be thrown out of employment, and that those who control improved methods will monopolize the business. The first objection is always urged against any labor-saving machine or important invention and improvement. But in this case, the men who have been employed in night scavenging will naturally find employment with those who introduce or adopt improved and acceptable apparatus. To the second objection it is answered that no single company or apparatus can monopolize the business of scavenging in this city, for the reason that the Board of Health would not refuse permits to any person or persons duly licensed by the Mayor, or who are able to remove night-soil "in such manner as shall prevent entirely the escape of noxious odors therefrom."

The effort to secure a great sanitary reform by the introduction of improved methods in night scavenging, is mainly in the interest of those who are obliged by circumstances to live in crowded tenements, and who are entitled by every consideration of humanity, as well as by public policy, to be protected, so far as possible, from everything that renders the air impure or their dwellings uncomfortable. While private dwellings are generally provided with water-closets connecting with street sewers, the houses of the poor and the laboring classes are only supplied with privies and sinks that must be frequently cleaned and emptied. The Board of Health would not be entitled to public confidence and respect if it failed to protect this part of the community from the foul and noxious odors of night scavenging. That the contamination of the atmosphere by such odors is unnecessary, has been demonstrated, and is no longer a theory or an experiment. In twenty of the largest cities of the United States the old system of scavenging has been discarded and prohibited by the proper authorities, and new and improved methods have been adopted. It is to be hoped that New York City will not be the last to adopt any and all improvements which will promote and secure the health and comfort of the people.

### WHISKEY FOR BABIES.

Dr. Jacobi, of this city, is a recognized authority in the diseases of children, and in their general care and management. In connection with Mary Putnam Jacobi he has lately published a valuable little work called "*Infant Diet.*" We were quite surprised to find therein a recommendation that nursing children should, in hot weather, take a little whiskey with their food and water. The quantity advised is small, not more than a teaspoonful in twenty-four hours. With all deference for so high an authority as Dr. Jacobi, we venture the opinion that that is a teaspoonful too much. Why a healthy baby should take whiskey with his food, especially *in hot weather*, is more than we can understand. It is well known that adult persons who use whiskey are more subject to the diseases which prevail in summer than are those who drink no liquor. We are inclined to hold to the old prescription, "milk for babes." Healthy infants need whiskey to keep them so, precisely as much as they need ether, or chloroform, or opium, or tobacco, or tea, or coffee; and they are better off without any of these stimulants.

The worst country is that in which we have no friend.

